# Role of macrophage scavenger receptor MSR1 in the progression of non-alcoholic steatohepatitis

**DOI:** 10.3389/fimmu.2022.1050984

**Published:** 2022-12-15

**Authors:** Wei Sheng, Guang Ji, Li Zhang

**Affiliations:** Institute of Digestive Diseases, Longhua Hospital, Shanghai University of Traditional Chinese Medicine, Shanghai, China

**Keywords:** NAFLD, MSR1, macrophage, lipid uptake, foam cell formation

## Abstract

Nonalcoholic steatohepatitis (NASH) is the progressive form of nonalcoholic fatty liver disease (NAFLD), and the dysregulation of lipid metabolism and oxidative stress are the typical features. Subsequent dyslipidemia and oxygen radical production may render the formation of modified lipids. Macrophage scavenger receptor 1 (MSR1) is responsible for the uptake of modified lipoprotein and is one of the key molecules in atherosclerosis. However, the unrestricted uptake of modified lipoproteins by MSR1 and the formation of cholesterol-rich foamy macrophages also can be observed in NASH patients and mouse models. In this review, we highlight the dysregulation of lipid metabolism and oxidative stress in NASH, the alteration of MSR1 expression in physiological and pathological conditions, the formation of modified lipoproteins, and the role of MSR1 on macrophage foaming and NASH development and progression.

## 1 Introduction

Nonalcoholic fatty liver disease (NAFLD) is characterized by lipid accumulation in the liver, and the disease spectrum ranges from simple steatosis (NAFL) to nonalcoholic steatohepatitis (NASH), and even cirrhosis and hepatocellular carcinoma (1). Due to the strong association between NAFLD and metabolic syndrome, the definition of metabolic-associated fatty liver disease (MAFLD) has been recently proposed (2). It is estimated that about 20% of patients with NAFLD will progress to NASH, and approximately 20-30% of NASH patients will develop liver fibrosis or even cirrhosis (3, 4). The continuous accumulation of lipids is a typical feature of NAFLD, and also an important risk factor for disease progression. In addition, dysregulated lipid metabolism correlates NAFLD to cardiovascular disease, a common cause of death in patients with NAFLD (5, 6). NAFLD is usually associated with dyslipidemia and oxidative stress, producing reactive oxygen species (ROS) might contribute to the modification of various lipids. Modified lipids are specifically transported by macrophage scavenger receptors (SRs), while the accumulation of modified lipids is the inducer of foam cells, as often observed in the pathogenesis of atherosclerosis and the liver of NASH (7, 8). Thus, SRs might be potential drug targets for NAFLD.

Liver is the major organ for lipid metabolism and immune surveillance, and contains a large number of immune cells including macrophages (9). Liver macrophages are divided into two distinct populations, liver resident macrophages from the fetal yolk sac called Kupffer cells (KCs) and monocyte-derived macrophages (MDMs) from the blood. These macrophages can mediate the clearance of apoptotic cells and foreign pathogens through phagocytosis and cytokine production (10). Notably, they are also the primary regulators of the inflammatory response. Depending on the microenvironment to which they are exposed, macrophage populations can transdifferentiate to different phenotypes, a process known as macrophage polarization. Both the plasticity and heterogeneity of macrophages determine their different phenotypes and functions (11–13). Macrophage is roughly divided into classically activated macrophages (M1 polarization) and alternatively activated macrophages (M2 polarization). M1-like macrophages obtain strong bactericidal and tumor-killing activity, whereas M2-like macrophages have efficient phagocytic activity, and are actively involved in tissue remodeling and repair as well as tumor progression (14, 15). Moreover, recent studies revealed that M2-like macrophages produce complex cytokines and may have various functions. Depending on different stimuli, M2-like macrophages can be further divided into M2a (induced by IL-4/13), M2b (induced by immune complex), M2c (induced by IL-10, transforming growth factor -β or glucocorticoids), and M2d (induced by agonists of toll-like receptors and adenosine A2 receptor) subtypes (16, 17).

KCs express specific surface markers such as F4/80^high^, CD11b^int^, and a high density of SRs, TLRs, Fc receptors, complement receptors, Nod-like receptor and mannose receptors. Monocytes or MDMs, on the other hand, express specific surface markers such as F4/80^int^ and CD11b^high^. In normal liver, KCs are the predominant liver macrophages, however, when NAFLD occurs, especially in the NASH state, lipotoxic hepatocytes stimulate KCs to release chemokines, such as CCL2, CCL5 and CSCL10, and render the hepatic recruitment of MDMs. The increase of macrophages that infiltrate around damaged hepatocytes is a typical feature of NASH (18, 19). Recent studies have identified different expression profiles of KCs versus MDMs during NASH. Ccr2 is enriched in MDMs, whereas Clec4f and Vsig4 are specifically expressed by KCs. In classifying subsets of KCs and MDMs, Su et al. found that the proportion of KC3 subsets with the chimeric expression of endothelial cells and KCs markers decreased from 11.78% in healthy livers to 3.39% in NASH livers, and these KCs tends to accumulate around injured hepatocytes, forming a distinctive coronal structure (18). Generally, KCs exhibit a stronger ability in regulating immune response, amino acid/carbon metabolism, and complement/coagulation cascade pathways, whereas MDMs show leukocyte chemotaxis and cell proliferation capacity.

**Figure 1:**
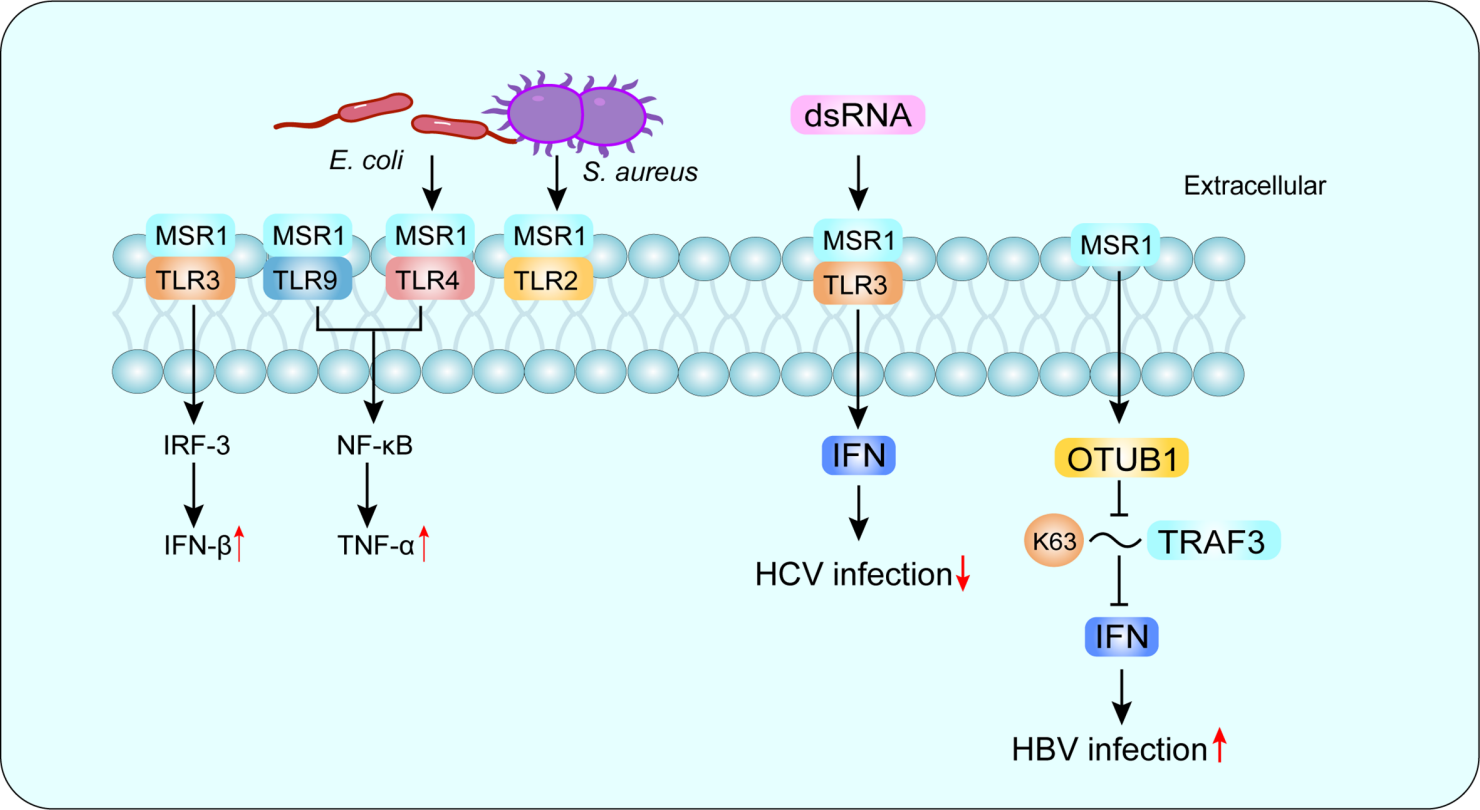
MSR1 in recognizing PAMP. MSR1 recognizes a large number of PAMP and plays an important role in host defense. MSR1 can also form co-receptor complexes with other receptors and mediate different or even opposite signaling pathways. For example, MSR1 and TLR4 complex activates phagocytosis of the Gram-negative bacterium *Escherichia coli*, while MSR1 interacts with TLR2 to promote phagocytosis of the Gram-negative bacterium *Staphylococcus aureus*. More interestingly, MSR1 promotes HBV infection and suppresses HCV infection. PAMP, pathogen-associated molecular patterns; TLR, Toll-like receptor; HBV, hepatitis B virus; HCV, hepatitis C virus.

SRs are defined as a class of cell surface receptors that can bind to multiple ligands and facilitate the clearance of non-self or altered self-components. In 2017, experts classified SRs in metazoan into 12 categories at a discussion forum organized by the United States National Institute of Allergy and Infectious Diseases, National Institutes of Health (20). Among them, the class A macrophage scavenger receptor (SR-A) is considered to be an innate immune pattern recognition receptor (PRR) that recognizes both damage-associated molecular patterns (DAMP) and pathogen-associated molecular patterns (PAMP) (21). Macrophage scavenger receptor 1 (MSR1) (also known as SR-AI, CD204 and SCARA1) is a typical class of SR-A molecules and the first SR to be cloned, originally isolated from bovine lung mRNA, and subsequently found in other species, including mouse and human (22). MSR1 is mainly expressed on macrophages and dendritic cells, and recent studies have found that MSR1 is also present on the surface of lymphocytes and may be involved in the pathogenesis of asthma and chronic obstructive pulmonary disease (23). Since macrophages mediate pathogen elimination through phagocytosis and cytokine production, MSR1 expression is important for the elimination of foreign pathogens (10). LPS is an important endogenous deleterious agent, and TLR4 is the main ligand of LPS, MSR1 and TLR4 cooperation can activate phagocytosis of the Gram-negative bacterium *Escherichia coli*, while MSR1 interacts with TLR2 to promote phagocytosis of the Gram-negative bacterium *Staphylococcus aureus* (24). Furthermore, MSR1 interacts with TLR4 and can signal through activation of NF-κB, which enters the nucleus and binds to DNA, leading to the synthesis of inflammatory cytokines (25) (**Figure 1**).

There are three isoforms of MSR1, with variable splice variants of the same identified gene, defined as SR-AI, SR-AII and SR-AIII (26–28). Other members of SR-A family include macrophage receptor with a collagen structure (MARCO), scavenger receptor A5 (SCARA5) and SR with a C-type lectin structural domain (SRCL-I/II), also known as the collector from the placental receptor (20). MSR1 recognizes a variety of ligands, including natural proteins, such as apolipoprotein (Apo) E; modified lipoproteins but not unmodified low-density lipoprotein (LDL), such as oxidized LDL (ox-LDL), acetylated LDL (ac-LDL) and modified type III collagen (20); polysaccharides, such as fucoidan; lipids, such as cholesterol; and other ligands, such as apoptotic cells, gram-negative and gram-positive bacteria (29). Depending on the recognized receptors, MSR1 can trigger different signaling pathways and mediate different or even opposite biological functions (30). In hepatitis C virus (HCV) infection, MSR1 binds to extracellular dsRNA, mediates endocytosis and translocation to endosomes through TLR3, and triggers a local antiviral response to limit HCV replication (31).While in hepatitis B virus (HBV) infection, MSR1 can directly interact with TNF receptor-associated factor 3 (TRAF3) and inhibit its ubiquitination associated with K63, and negatively regulate TRAF3 protein stability and attenuate the innate immune response of type I IFN to HBV infection by promoting the recruitment of OTUB1 to TRAF3 (32) **(** **Figure 1** **)**. Compared with other SR-A family members, MSR1 is more efficient in promoting the degradation of modified LDL, which may affect the subsequent cholesterol deposition in cells (33). In the liver, MSR1 is enriched in KCs at a physiological state (33–35). In addition, MSR1 mediates the uptake of lipids in KCs and triggers liver inflammation in the development of NASH (8). Therefore, MSR1 integrates lipid metabolism and immune regulation in the liver, and understanding its functions may contribute to NASH prevention and therapy.

## 2 MSR1 accelerates NAFLD progression

The pathogenesis of NASH development and progression is complex, and the most popular hypothesis is the “parallel multi-hits” theory, which is based on the accumulation of lipids in the liver due to insulin resistance. Overwhelming lipid supply and fatty acid oxidation cause subsequent oxidative stress and liver injury; simultaneously, cytokines from adipose tissue, endotoxins and certain metabolites derived from the intestine all contribute to the development and progression of NASH (36, 37). Lipid accumulation and oxidative stress commonly coexist in NAFLD (38), and liver macrophages (both KCs and recruited macrophages) respond to the altered metabolic pattern and accelerate the progression to NASH (39). Increased macrophage infiltration in the liver has been reported to be the initiator of NASH (40). Macrophages are innate immune cells that constitute the front line of host defense against pathogens. It has been shown that inflammatory activation of macrophages also induces the dysfunction of lipid metabolism (41, 42). Recent data suggest that the vast majority of macrophages in the healthy liver are KCs (43), but in NASH, MDMs outnumber KCs and become the dominant macrophage subpopulation in the liver (44). In NASH patients and mice, macrophages typically accumulate in clusters around the steatotic hepatocytes and can promote the progression of NAFL to NASH (44, 45). In addition, a high-fat/cholesterol/cholic acid diet mouse model that assembles NASH-associated fibrosis also showed that liver fibrosis is initiated around lipid-associated macrophages (46). More interestingly, Anneleen et al. found that during the progression of NAFLD, liver resident KCs are gradually lost, which might be attributed to the alteration of the transcriptome and proliferation in the niche cells during NAFLD progression. Moreover, signals that maintain the development of KCs are also destroyed along with the morphological changes in hepatocytes during NAFLD (19). Meanwhile, MDMs are recruited into the liver in response to inflammatory responses, and these recruited macrophages are confined to the portal and central vein, the same location that is observed for KCs in a healthy liver. These recruited macrophages further differentiate into different subsets, and macrophages expressing certain KC signature genes (e.g., Clec4f) are defined as monocyte-derived KCs, monocyte-derived KCs lack expression of other KC-specific genes (e.g., Timd4), thus function differently from resident KCs (47). Liver resident KCs are previously considered to be pro-inflammatory in NAFLD (48, 49), however, some recent studies have questioned this view and demonstrated that recruited monocyte-derived KCs are the real culprit responsible for the development of liver inflammation and fibrosis (19, 50, 51). Interestingly, ultrastructure imaging revealed that the resident KCs exhibit multiple small, electron-dense liposomes, whereas the monocyte-derived KCs demonstrate a larger liposome with an electron-lucent center (50). Moreover, a subset of lipid-associated macrophages that are derived from monocytes are activated in response to stimuli and have different lipid metabolism abilities, which may be highly related to liver fibrosis (19). Because many of the existing studies have examined the function of KCs through their non-specific depletion or non-specific markers, the roles of KCs in NAFLD are often conflicting, and the relative contributions of resident KCs and recruited MDMs remain largely unknown (14, 19). Subsets classification of KCs and recruited MDMs by more precise and stable markers would help clarify the exact roles in NAFLD.

Under physiological conditions, LDL transports and secrets cholesterol-containing particles to the circulation, whereas HDL transports cholesterol in reverse and is responsible for transporting circulating cholesterol back to the liver. LDL receptor (LDLR) is one of the endocytic receptors and a key receptor for the clearance of plasma cholesterol. LDLR distributes in the cell surface of the sinusoidal region and binds to and internalizes circulating LDL and other lipoproteins containing ApoB-100 and ApoE. LDLR is abundantly expressed in the liver, where it is a key receptor in maintaining the homeostasis of lipid metabolism in mammals (52). LDL is the most important physiological ligand of LDLR, but once LDL is modified (e.g., ox-LDL), it is taken up by MSR1, cluster of differentiation 36 (CD36) and lectin-like ox-LDL receptor-1 (LOX-1) on the surface of macrophages (53). Studies have shown that MSR1, CD36 and LOX-1 on the macrophage surface are collectively responsible for approximately 90% of ox-LDL uptake (53, 54).

In general, MSR1 is almost equally abundant on unpolarized, alternatively and classically activated macrophages, but its abundance is increased on phagosomes of activated macrophages (55). In liver samples of NAFLD patients, MSR1 is predominantly expressed on KCs and mature foamy macrophages, rather than infiltrating MDMs. MSR1 protein expression is increased with disease progression, especially when monocytes are differentiated into macrophages (8, 56). MSR1 transcript levels are significantly associated with the incidence of hepatic steatosis, cirrhosis, and hepatocellular carcinoma in patients with NAFLD; and MSR1 protein expression also increases with disease progression (8). In addition, mRNA and protein expression levels of MSR1 and CD36 are upregulated after the artificial treatment of RAW264.7 cells with ox-LDL (53). This evidence suggests that excessive extracellular free fatty acids (FFAs) may induce the increased expression of MSR1 to handle excessive environmental lipids. Notably, soluble MSR1 can also be identified, which has been shown to promote inflammatory responses in Th17 cells and exacerbate disease progression (57). However, current studies have not yet clarified whether soluble MSR1 is produced through cleavage from cell-associated MSR1 or through some other molecular mechanisms (29).

**Figure 2:**
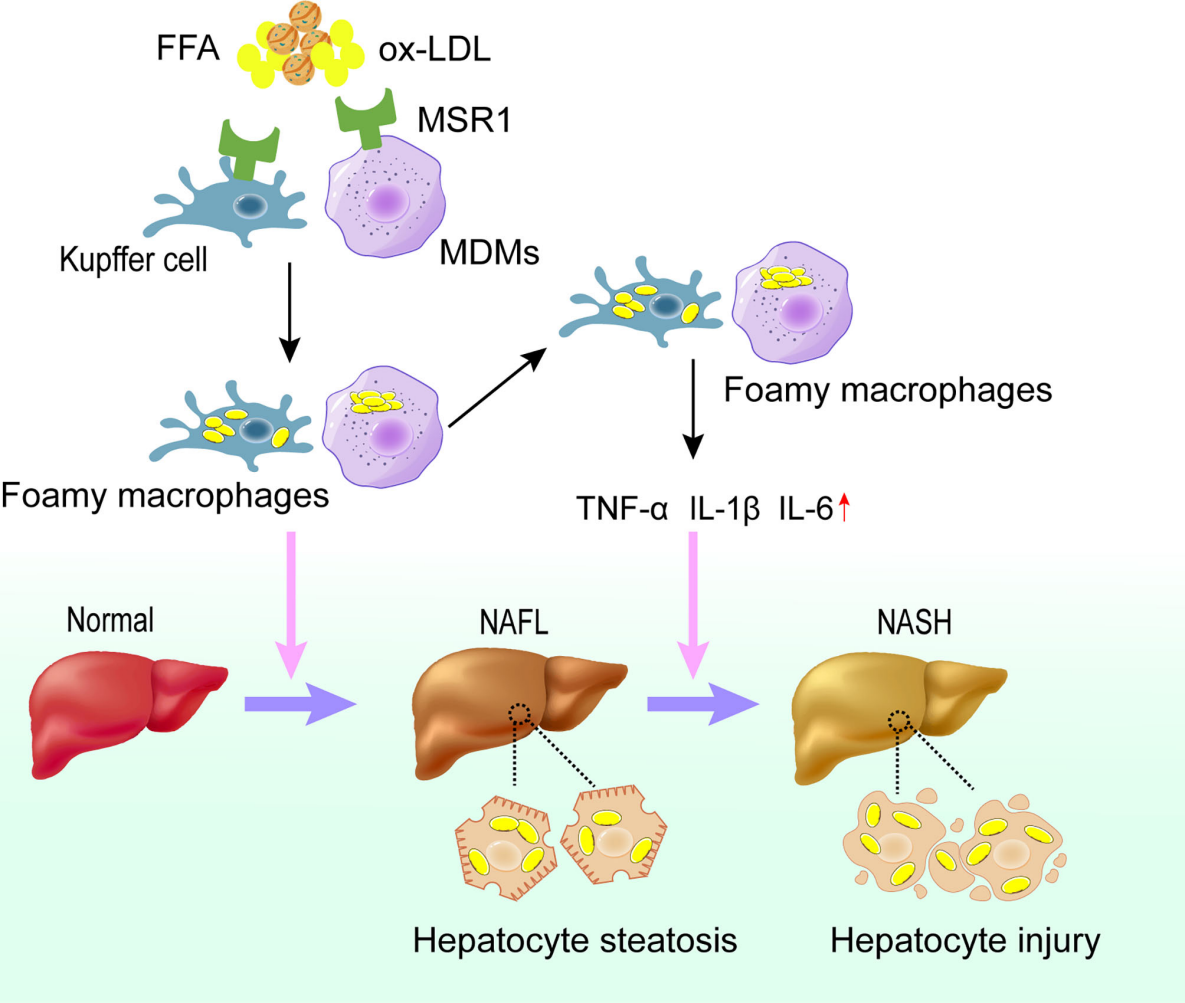
Role of MSR1 in NASH development and progression. In the early stage of NAFLD, dysregulated lipid metabolism promotes the expression of MSR1 in Kupffer cells and MDMs. MSR1 is responsible for the uptake of modified lipoprotein, leading to the formation of foamy macrophages and lipid accumulation in the liver. Meanwhile, foamy macrophages can promote the release of inflammatory factors and aggravate the injury of hepatocytes, thus promoting the progression of NASH. NAFL, simple steatosis; NASH, nonalcoholic steatohepatitis; MDMs, monocyte-derived macrophages.

Collectively, macrophage infiltration in the liver of NAFLD patients as well as the elevated levels of its surface receptor MSR1 significantly promote the uptake of lipids, which may further induce the formation of foamy cells and promote the progression into NASH **(** **Figure 2** **)**.

## 3 Disturbed lipid metabolism and oxidative stress up-regulate MSR1

Simple steatosis without hepatocellular injury is usually considered benign, and the presence of lobular inflammation and hepatocyte injury indicate the progression to NASH (38, 58). However, the underlying mechanisms of the disease progression are largely unknown. FAs are composed of non-esterified FAs (e.g., saturated and unsaturated FAs) and modified FAs (e.g., oxidized, halogenated, or otherwise modified FAs), and their sources include dietary-derived chylomicron and *de novo* lipogenesis in the liver (59). FAs provide energy through β-oxidation in mitochondria, β-oxidation in peroxisomes and ω-oxidation in microsomes, but in NAFLD, lipid overload and insulin resistance lead to an overflow of FAs to the liver, and mitochondria enhance β-oxidation and trigger excessive oxidative phosphorylation to eliminate these FAs. However, the process of adenosine triphosphate (ATP) generation through oxidative phosphorylation in the mitochondrial respiratory chain is also one of the main sources of ROS (60, 61). Because the oxygen molecule is an ideal terminal electron acceptor, it is easy to get transferred electrons during oxidative phosphorylation and then form ROS, which is a well-known oxidant with high chemical reactivity. The formation of ROS is mainly related to the overwhelming β-oxidation of FAs in mitochondria (62). Therefore, mitochondrial damage may increase ROS production, and excessive ROS is toxic to the mitochondria. M1-like macrophages activated by the classical pathway also produce large amounts of nitric oxide and ROS intermediates (63). ROS production and subsequent oxidative stress have been reported to play a key role in the pathogenesis of NAFLD (64). In addition, oxidative stress can exacerbate lipid accumulation in hepatocytes, further promoting ROS production (60, 61). It is reported that ROS activates NLRP3 and induces inflammatory vesicle downstream complexes through cystein-1, and initiates the release of pro-inflammatory cytokines such as IL-1β and IL-18, which further contributes to hepatocyte necrosis and the development of liver fibrosis (65). These findings seem to be consistent with clinical studies, as NAFLD patients have higher levels of both ROS and lipid peroxidation products than healthy controls, while lower levels of antioxidant enzymes, such as superoxide dismutase (SOD) and catalase (CAT), and lower levels of antioxidant compounds, such as glutathione (GSH) (66, 67). However, it is important to note that ROS-induced activation of the NF-κB pathway can promote phagosome maturation, suggesting that a large amount of ROS produced during macrophage activation is essential for the elimination of microbial pathogens (68, 69).

Oxidative stress is also one of the important mechanisms for liver injury during NAFLD, which is accompanied by mitochondrial dysfunction and endoplasmic reticulum stress, among others. The increased ROS production may target the double bonds of polyunsaturated FAs (PUFAs) and generate oxidized FAs *via* various complex enzymatic and non-enzymatic pathways (70). PUFA-containing lipids are usually the main targets of ROS attack, leading to their non-enzymatic oxidation and formation of extremely reactive aldehyde components, which induce hepatocyte damage, a process that is called lipid peroxidation (LPO). Under an oxidative stress state, PUFAs-containing phospholipids and cholesterol esters in cell membranes and lipoproteins are readily oxidized by the free radical-induced LPO process, forming a complex mixture of oxidation products (71). For example, a large number of oxygen radicals generated by mitochondria can oxidize and break PUFA double chains of LDL, which are then cross-linked to form conjugated dienes with ApoB, thereby altering the surface structure of LDL and forming ox-LDL. Ox-LDL is chemically unstable and readily reacts with surrounding tissues. The main response pathways that are involved in liver inflammation in western diet-fed LDLR-deficient mice are related to biological processes such as innate immunity and oxidative stress, and both *in vitro* and *in vivo* experiments demonstrated that malondialdehyde stimulates cytokine secretion as well as leukocyte recruitment, and this malondialdehyde-induced cytokine secretion is dependent on MSR1 and CD36 (72) (**Figure 2**).

**Figure 3:**
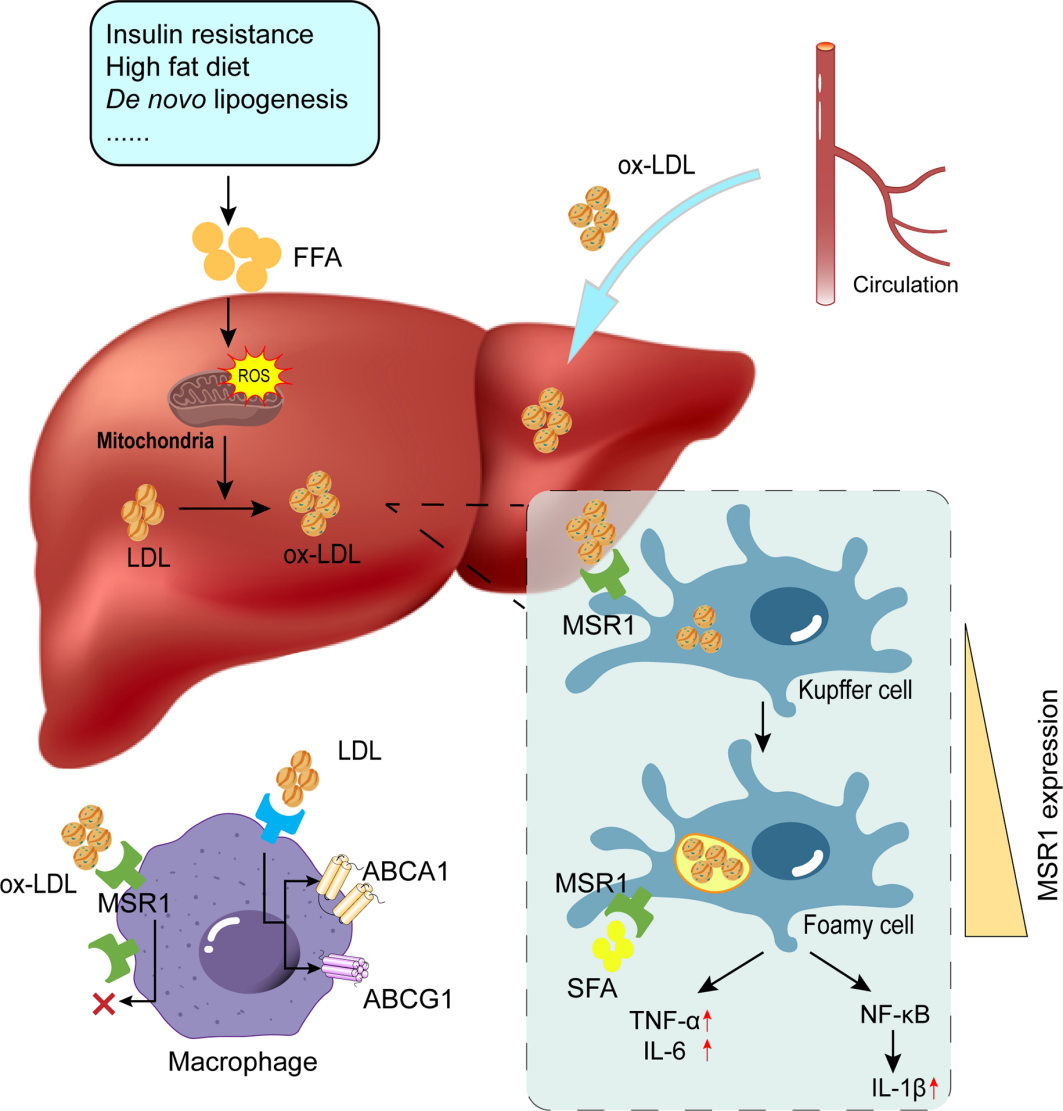
Role of MSR1 in foamy cell formation. Due to factors such as insulin resistance and overnutrition, the occurrence of lipid accumulation and subsequent ROS production promotes the conversion of modified lipids (e.g., ox-LDL and ac-LDL). MSR1 is primarily responsible for ox-LDL uptake. With unrestricted uptake of ox-LDL and limited lipid efflux, KCs will convert into foamy cells. Concomitantly, MSR1 expression on the cell surface is also up-regulated upon various stimulation. ROS, reactive oxygen species; LDL, low-density lipoprotein; ox-LDL, oxidized low-density lipoprotein; KCs, Kupffer cells.

NASH is associated with disturbed lipid metabolism and excessive oxidative stress in the liver. Mitochondria regulate lipid metabolism, mitochondrial dysfunction can lead to disruption of lipid homeostasis and oxidative stress, which are major contributors to NASH (73–75). Elevated levels of ROS and LPO products, a vicious cycle of ROS and oxidative stress, and mitochondrial dysfunction significantly contribute to ox-LDL production and subsequent MSR1 elevation (64). In addition, oxidative stress in NAFLD has been associated with peroxisomal β-oxidation, endoplasmic reticulum stress, and xanthine oxidase (76–78). Damaged cells release DAMPs, which bind to TLR4 or TLR9 on KCs to activate the NF-κB signaling pathway, thereby generating more ROS to amplify the inflammatory response (64) **(** **Figure 3** **)**. Accordingly, antioxidants such as silymarin or silybin, resveratrol, hexoketococine, and vitamins A, C, and E, are potential agents against NASH (79).

## 4 MSR1 contributes to macrophage foaminess

The intracellular cholesterol esters and free cholesterol are in dynamic equilibrium in macrophages. Under physiological conditions, only unesterified free cholesterol can be transported into macrophages, and excess intracellular cholesterol esters are stored as lipid droplets. When the intracellular free cholesterol level is too high and the cells are unable to eliminate excess cholesterol, free cholesterol will be esterified in the endoplasmic reticulum to reduce the cytotoxicity. Acyl-coenzyme A: cholesterol acyltransferase 1 (ACAT1) and neutral cholesteryl ester hydrolase (nCEH) are involved in maintaining intracellular lipid homeostasis in macrophages (80). Disturbance in intracellular lipid metabolism in macrophages is a prerequisite for the formation of foam cells. Under normal conditions, a dynamic balance is maintained between lipid uptake, synthesis, esterification, hydrolysis and efflux in macrophages, but altered extracellular lipids can indirectly lead to disruption of intracellular metabolism, with altered expression of receptors and enzymes related to lipid metabolism in macrophages (**Figure 2**).

Under physiological conditions, LDLR binds to LDL and takes up cholesterol esters into cells for cell proliferation, steroid hormone synthesis and bile acid salt synthesis. However, when LDL is modified (e.g., ox-LDL, ac-LDL), SRs are primarily responsible for internalizing LDL into macrophages. Studies have shown that MSR1, CD36 and SR-B1 are the main SRs that mediate ox-LDL clearance (81–83). Compared to quiescent macrophages, macrophages activated by IFN-γ and TNF-α express less MSR1 and CD36 proteins on their surface and therefore have lower cholesterol accumulation after treatment with ox-LDL (84). In addition, the uptake of ac-LDL was reduced by approximately 80% in both human MDMs and PMA-treated THP-1 macrophages after silencing MSR1, indicating that MSR1 is also involved in the uptake of ac-LDL (85). Another study reported that perivascular adipose tissue-derived exosomes can reduce macrophage-derived foam cell formation by downregulating MSR1 expression (86). These studies suggested that MSR1 mediates the uptake and degradation of modified LDL, leading to substantial intracellular cholesterol deposition, and plays a key role in macrophage foaminess (87).

Accumulation of cholesterol in cells activates the cholesterol sensor liver X receptor (LXR). Activation of LXR in macrophages induces genes involved in reverse cholesterol transport, including ATP-binding cassette transporter A1 (ABCA1) and ATP-binding cassette transporter G1 (ABCG1). In addition, LXR activation can also promote the degradation of SREBP-1c and β-hydroxy-β-methylglutaryl CoA reductase, thereby limiting cholesterol biosynthesis (9). Thus, under normal conditions, the accumulation of intracellular cholesterol activates LXR, which promotes its clearance and limits the synthesis of cholesterol. However, ox-LDL can induce physical changes in the membrane or cytoskeleton of macrophages, leading to significant biomechanical changes that affect cellular behavior (88). Upon ox-LDL treatment, MSR1 and CD36 in macrophages are upregulated to enhance ox-LDL uptake (53). ox-LDL/SR pathway is thought to be the main mechanism of macrophage-derived foam cell formation (89). To date, MSR1 is known to be one of the major contributors to ox-LDL uptake, and its role in atherosclerosis has been extensively studied. In the early stages of atherosclerosis, cholesterol induces oxidative stress on the walls of blood vessels and promotes LDL modification, then modified LDL activates endothelial cells to release adhesion molecules, inflammatory factors, and chemotactic monocytes migrate to the subendothelial layer and differentiate into macrophages, whose surface protein MSR1 binds and internalizes modified LDL(e.g., ox-LDL and ac-LDL), resulting in lipid deposition in macrophages and eventually cholesterol-rich foam cells (90). The release of inflammatory factors aggravates endothelial cell injury and increases monocyte chemotactic protein-1 expression, promoting cholesterol deposition in macrophages through upregulating MSR1 (91); at the same time, MSR1 overexpression on macrophages also promotes macrophage adhesion and subendothelial retention (92).

Similarly, elevated MSR1 levels in the liver also mediate the uptake and degradation of modified LDL, leading to the deposition of cholesterol-contained cytoplasmic droplets, resulting in the formation of foamy macrophages (87). Intracellular free cholesterol is normally translocated to the endoplasmic reticulum, but when cholesterol is overloaded, it will trigger endoplasmic reticulum stress and ultimately leads to cell apoptosis (93). In addition, ox-LDL can also induce a pro-inflammatory phenotype of macrophages in an MSR1-dependent manner (94) **(** **Figure 3** **)**. As MSR1 levels are increased in visceral adipose tissue in obesity, further *in vitro* experiments of visceral adipocytes demonstrated that MSR1-mediated increase of ox-LDL uptake stimulates the pro-inflammatory responses (95). It has been reported that cholesterol uptake by MSR1 is generally unrestricted, and interestingly, one study found that MSR1 uptake of ox-LDL promotes the stabilization of its mRNA; the mechanism might be attributed to the fact that ox-LDL promotes the expression of the DEAD-box protein 5 (DDX5, an ATP-dependent RNA helicase) in macrophages, which interacts with Mettl3 to stabilize the mRNA of the MSR1 by reducing the m6A modification (96). More importantly, MSR1 expression was upregulated after LPS stimulation in macrophage RAW264.7, accompanied by increased foaming of macrophages and increased levels of inflammatory factor (97, 98).

Previous studies have shown that *MSR1* expression decreased in a dose-dependent manner after treatment with cranberry extract (99). Nobiletin isolated from tangerines, and Zerumbone extracted from *Zingiber zerumbet* (L.) Smith specifically can also inhibit ac-LDL uptake by macrophages *via* mediating MSR1 expression (100, 101). Formononetin extracted from *Astragalus membranaceus* can increase the expression of Krüppel-like factor 4, which acts as a transcription factor and negatively regulates MSR1 expression, thereby inhibiting foam cell formation (102).

## 5 MSR1 in regulating metabolic inflammation

In chronic liver inflammation, MSR1 transcript levels are significantly correlated with the incidence of hyperlipidemia, cirrhosis and hepatocellular carcinoma (8). This is because MSR1 accelerates lipid uptake in KCs and triggers hepatic inflammation (8, 103). Saturated FAs can induce hepatocyte apoptosis. It is found that MSR1 promotes the uptake of saturated FAs by macrophages, and saturated FAs induce increased transcript of *Tnf-α* and *Il-6* through the JNK-mediated pathway. In contrast, in the liver and white adipose tissue of *Msr1^-/-^* mice, the expression of *Tnf-α* and *Il-6* is decreased and the serum levels of IL-6 and TNF-α are lower (8). Notably, *Msr1^-/-^* mice appear to be protected from high-fat and high-sugar diet-induced metabolic disturbances, the mice exhibit fewer hepatic foamy macrophages, less hepatic inflammation, improved dyslipidemia and glucose tolerance, and altered hepatic lipid metabolism. Interestingly, Zhu et al., on the other hand, found that obese *Msr1^-/-^* mice exhibit increased insulin resistance and enhanced inflammation characterized by polarization of macrophage populations toward pro-inflammatory subpopulations. The possible explanation is that the deletion of MSR1 inhibits the conversion of macrophages from M1 to M2 in adipose tissue, thus increasing insulin resistance in mice (104). Similarly, Cavallari et al. found that deletion of the *Msr1* or treatment with fucoidan, a natural product antagonistic ligand of MSR1, both worsened white adipose tissue insulin resistance in HFD-feeding mice (105). And notably, insulin is reported to reduce *Msr1* expression in human macrophages (106). These reports suggest that *Msr1* deletion exacerbates HFD-induced insulin resistance during diet-induced obesity in mice. However, studies on the MSR1 antagonistic ligand fucoidan seem to be inconsistent, since Goldstein et al. first demonstrated that fucoidan binds to MSR1 at the LDL recognition site of macrophages (107), numerous studies have found that fucoidan can lower blood glucose and improve insulin resistance, steatosis, endoplasmic reticulum stress and inflammation in obese mice and rats (29, 108–110). This contradicts the findings that fucoidan worsens insulin resistance in HFD-fed mice, and upregulates TNF-α production in J774A.1 cells (105, 111). Seimon et al. found that MSR1 binds antagonistically to fucoidan, interacts with TLR4, and triggers JNK-dependent apoptosis in ER-stressed peritoneal macrophages (112). In addition to interacting with other receptors or pathways, fucoidan can inhibit LPS and polyanionic polypeptide internalization into J774 macrophages *via* MSR1 (29). In sepsis studies simulated with LPS, *Msr1^-/-^* mice survived at a lower rate than wild type mice, and this protective effect is associated with the anti-inflammatory effect of MSR1 in dendritic cells (113). In another LPS-induced sepsis model, MSR1^+^ monocytes and/or macrophages also inhibit the acute inflammatory response by suppressing the upregulation of TNF-α and IL-6 levels (56). Paradoxically, Kobyashi et al. observed that *Msr1^-/-^* mice survive at a higher rate than wild-type mice after LPS treatment because of reduced clearance of LPS and lower serum levels of IL-1β, indicating that a lack of macrophage surveillance in the absence of MSR1 (114).

MSR1 is reported to trigger the JNK-mediated pro-inflammatory phenotype of macrophages. IL-4- activated macrophages normally stimulate the activity of the nuclear receptor peroxisome proliferator-activated receptor γ (PPARγ), which has been shown to mediate alternative macrophage activation as well as transcriptional repression of several pro-inflammatory factors (84). However, triggering MSR1 in IL-4-activated macrophages leads to K63 polyubiquitination of MSR1 in macrophage phagosomes, which enhances recruitment of the TAK1/MKK7/JNK complex to the phagosomes, which may facilitate the transition of macrophage from the M2 anti-inflammatory state to the M1 pro-inflammatory state (94). Furthermore, triggering MSR1 in this model does not activate the NF-κB pathway; however, another study showed that in spinal cord injury, particularly in the presence of myelin debris, MSR1 promotes phagocytosis of myelin debris, foam macrophage formation, and the release of the inflammatory mediator IL-1β through mediating the NF-κB signaling pathway (115). Conversely, some studies have shown that MSR1 also acts as an inhibitor of inflammation under certain circumstances, regulating early neutrophil recruitment by downregulating the production of chemokines from resident peritoneal macrophages (116). Zhao and his team found that the MSR1-activated PI3K/AKT/GSK3β/β-linked protein pathway targets the proliferator-activated receptor γ coactivator 1-α and promotes M2-like polarization by enhancing mitochondrial oxidative phosphorylation (117). MSR1 is highly expressed in macrophage clusters and may be involved in regulating M2-like macrophage polarization, knockdown of *Msr1* inhibits M2-like macrophage polarization, and elevated MSR1 expression promotes macrophage polarization toward the M2 type (118, 119).

Although these results are contradictory, it is undeniable that triggering MSR1 may lead to alterations of macrophage phenotype and the type of produced cytokines, probably due to different MSR1 ligands may trigger different signaling cascades. MSR1-mediated signaling depends on the binding and internalization of MSR1 to different ligands, and MSR1 binding to different ligands often leads to completely different or even opposite signals (55). In addition, MSR1 can affect signal transduction by forming different complexes with co-receptors. It should be noted that MSR1 receptors might be unable to bind higher affinity ligands due to preexisting low-affinity ligands, which are referred to as non-mutually beneficial cross-competition. Therefore, the order in which MSR1 binds its ligand may also influence signal transduction (120). Collectively, these might be the possible reasons for the current controversial findings of MSR1 studies.

## 6 MSR1 in the hypoxic environment

Hypoxia-inducible factor (HIF)-1α is a major stimulator of oxygen-regulated gene expression. Earlier studies have shown that hypoxia facilitates lipid droplet accumulation and that HIF-1α knockdown inhibits foam cell formation (121, 122). LXR expression is reported to be induced in human macrophages and RAW264.7 cells under hypoxic conditions; however, while LXR activation promotes reversal cholesterol transport, it also increases the stability of HIF-1α protein and synergistically induces lipid accumulation in macrophages within atherosclerotic plaques (123). In patients with obesity, increased intracellular FFA stimulates adenine nucleotide translocase (ANT)2, leading to increased oxygen consumption and producing a state of relative hypoxia. This triggers the production of HIF-1α-dependent chemokines, leading to adipose tissue inflammation, insulin resistance, and metabolic dysfunction (124–126). ANT2 triggers ROS production and mitochondrial damage and leads to pro-inflammatory macrophage activation (127). In addition, it has been demonstrated that the HIF-1-PTEN/NF-κB-p65 pathway plays an important role in NAFLD progression; and in NAFLD mouse liver and HepG2 cells, HIF-1α overexpression and PTEN low expression both activate the NF-κB-p65 pathway and exacerbate the inflammatory response (128). This evidence suggests that hypoxia can promote lipid accumulation and pro-inflammatory activation of macrophages, leading to adipose tissue inflammation, insulin resistance and metabolic inflammation.

Further studies suggested that increased lipid uptake by macrophages with hypoxia is associated with the regulation of ox-LDL-related receptor expression (53). As previously described, MSR1, CD36 and Lox-1 are the receptors in responsible for transporting ox-LDL. Under normoxia, ox-LDL upregulates the expression of MSR1 and CD36 in RAW264.7 cells, but their levels decrease under hypoxic conditions. In contrast, Lox-1 expression is upregulated under hypoxic conditions (53). Thus, Margot and his colleagues suggested that Lox-1 plays a major role in hypoxia-induced foam cell formation. Furthermore, overexpression of HIF-1α in RAW264.7 cells under hypoxic conditions also suppresses the expression of MSR1 and reduces both the transcriptional activity of the MSR1 gene and the phagocytosis of the Gram-positive bacterium *Listeria monocytogenes* (129). Other studies also showed a decrease of MSR1 expression in hypoxic macrophages (130, 131). However, in a study using high-density oligonucleotide microarrays to investigate hypoxia-induced changes in human monocytes, MSR1 expression is found to be significantly elevated (132). Analysis in macro-array targeting angiogenic gene expression also revealed that hypoxia could induce an approximately a 30-fold increase of MSR1 expression in human microvascular endothelial cells (HMEC-1) (133). More importantly, hypoxia increases the expression of MSR1 and inflammatory cytokines in adipose tissue of morbid-obese patients, and HIF-1α silence suppresses MSR1 expression and adipose inflammation (95).

## 7 Conclusion and perspectives

As an important PRR on the surface of macrophages, MSR1 plays an important role in innate and adaptive immune regulation. To date, anti-MSR1 blocking antibodies have been developed that significantly inhibit TNF-α, IL-6 and IL-8 production and are used for related diseases (134). In addition, MSR1 is also a member of the SR family, which contributes to the phagocytosis of pathogenic microorganisms and self-components such as modified LDL and cellular debris. While previous studies have focused on elucidating its role in atherosclerosis, researchers have begun to focus on its possible contribution to NASH, elucidating the mechanism of unrestricted uptake of MSR1 for lipids in NASH development and progression. In addition, MSR1 is an indispensable protein, MSR1 is also involved in preventing the deposition of calcified particles due to its ability to remove calmodulin complexes in circulation (135).

Notably, the wide range of MSR1 ligands and the ability of MSR1 to cooperate with other different PRRs on the macrophage surface potentiate MSR1 in triggering different signaling pathways and performing completely different or even opposite biological functions (*e.g.*, pro-inflammatory or anti-inflammatory). Therefore, further studies are needed on MSR1, which might provide a new strategy for preventing NASH and its complications.

## Author contributions

LZ conceptualized the manuscript, WS collected the literature and drafted the manuscript, LZ and GJ revised the manuscript. All authors edited, revised, and approved the final version of this review.
